# More Than Only Skin Deep: Appearance Self-Concept Predicts Most of Secondary School Students’ Self-Esteem

**DOI:** 10.3389/fpsyg.2016.01568

**Published:** 2016-10-18

**Authors:** Tanja G. Baudson, Kira E. Weber, Philipp A. Freund

**Affiliations:** ^1^Department of Educational Sciences, Institute of Psychology, University of Duisburg-EssenEssen, Germany; ^2^Institute of Psychology, Leuphana University LüneburgLüneburg, Germany

**Keywords:** appearance self-concept, academic self-concept, social self-concept, multidimensional self-concept, self-esteem, adolescence

## Abstract

One important goal of education is to develop students’ self-esteem which, in turn, hinges on their self-concept in the academic, physical, and social domains. Prior studies have shown that physical self-concept accounts for most of the variation in self-esteem, with academic and social self-concepts playing a much lesser role. As pressure toward perfection seems to be increasing in education, appearance, and social relationships (three aspects that relate to crucial developmental tasks of adolescence), the goal of the present field study was to examine whether former findings still hold true in the light of the changing societal context. A sample of 2,950 students from a broad range of German secondary schools (47% girls, age 10–19 years) responded to a recently validated German-language questionnaire assessing multiple self-concept facets (Weber and Freund, 2016). We examined which self-concept aspects predict self-esteem best and whether the pattern is comparable across genders and achievement levels using latent regression analyses. Results show that self-concept of appearance is still by far the strongest predictor (total sample: *B* = 0.77, *SE* = 0.02, *p* < 0.01) and that this is especially the case for girls and students from special educational schools. Other aspects play a much lesser role. The discussion explores why appearance is so neglected, compared to the more academic subjects, and what school can do to account for its vast importance for students’ self-esteem.

## Introduction

**FIGURE 1:**
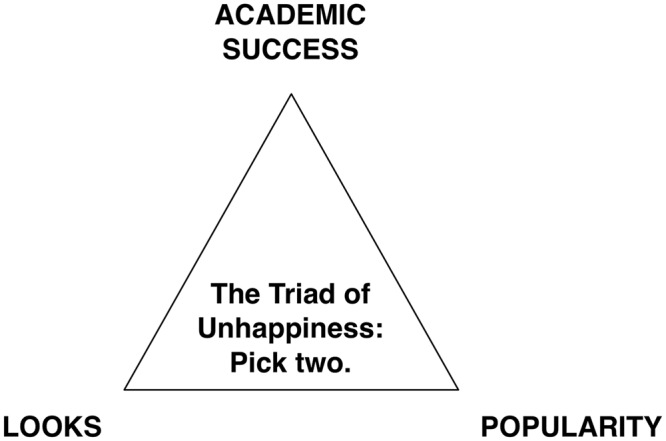
**The Triad of Unhappiness.** The figure represents an application of the well-known Triple Constraint Model from project management (originally comprising scope, time, and cost) to self-concept.

Looking at the representation of people in the popular media, one quickly gets an impression of the features of a perfect human being: success, good looks, and popularity—an ideal that, as a whole, is quite impossible to attain, leading to feelings of inadequacy if one identifies with these ideals (see **Figure 1** for a not-too-serious illustration of the Triad of Unhappiness). In line with Cooley’s (1902) “looking-glass self”—the assumption that one’s self-perception mirrors how one is perceived by others—it is merely logical that these ideal norms are reflected in the structure of self-concept (SC) as well. SC can be described as the beliefs and attitudes people hold about themselves. It is characterized by a hierarchical, multidimensional structure and thus comprises several facets of the self, which become increasingly fine-grained the further one descends in the hierarchy (Shavelson et al., 1976; Marsh and Roche, 1996). Usually, three main dimensions of SC are distinguished: academic (beliefs about one’s abilities, e.g., in mathematics or English), physical (the beliefs one holds about one’s appearance and physical abilities), and social (beliefs about relationships with others, e.g., peers, parents, or teachers), each of which can be split into even finer facets. General SC at the top of the apex represents the overall evaluation of oneself as a person and has often been used interchangeably with the term “self-esteem” (Shavelson et al., 1976)^1^.

### The Relationship between SCs and Self-Esteem

#### Mechanisms

One fundamental tenet of self-esteem research is that people strive to maintain a positive evaluation of themselves. As individuals differ in domain-specific strengths, they also differ with regard to which domains they consider important for their self-esteem and which they discard. According to James (1890/1963), failing in subjectively unimportant domains hardly affects self-esteem, whereas failure in important domains does (cf. Harter, 1993). Marsh (1986) contrasts James’ assumption to Rosenberg’s (1965) interactive hypothesis, which posits that the effects of a positive (or negative) SC on self-esteem are exacerbated when the domain in question is important, and more or less neutral when the domain is deemed unimportant. One strategy to dealing with failure is discounting the importance of the domains one is bad at and emphasizing those where one’s strengths are. However, this approach has its limitations, as it is hard to completely escape the values of the group(s) one belongs to.

The latter aspect is important in our context, too. First, societal changes are likely reflected in the importance of the different SC facets; second, differential expectations based, e.g., on gender, may affect their relative importance; and finally, the values students are confronted with in school furthermore influence which SC facets are deemed important and which are not.

The school context is crucial here, as the importance of a given domain depends not only on criterial norms (e.g., absolute beauty standards, graduating from the highest-tier secondary school), but also hinges very much on one’s relative position within one’s group, i.e., social norms. For adolescents, their classroom is a highly salient frame of reference, as they encounter their classmates almost daily and mutually shape each others’ perceptions of reality. Therefore, even if one does not conform to absolute standards, one’s self-esteem may still benefit from being the smartest, most popular, or most handsome kid in class. To paraphrase a common joke: one need not be faster than the lion, but only faster than the other person also fleeing from it.

Of note, more distinctive SC facets with greater variability contribute more to self-esteem than less distinctive facets (Marsh, 1986). This is important in school systems where tracks are formed based on academic achievement, like in Germany. Because students attending comprehensive schools are highly diverse with regard to their performance levels (compared to students from tracked schools), their academic SCs might exhibit greater variability as well.

Finally, besides criterial and social norms, the individual norm may affect self-esteem in at least two ways. First, the absolute level of SC depends on how different SC facets relate to each other. Dimensional comparison theory (DCT; Möller and Marsh, 2013) posits that SC discrepancies are particularly high between subjects that are perceived as dissimilar. Expanding on this notion, a similar “compensation” can be expected at least between academic and social self concept (drawing, e.g., on Cuddy et al.’s (2008) BIAS map, which showed competence and warmth to be independent dimensions in social perception, thus highly dissimilar). Further evidence comes from students attending gifted programs, who exhibited lower physical and social SCs than non-identified gifted adolescents, which suggests a perceived incompatibility between academic SC and the other two, assuming that academic achievement becomes more salient for students attending a gifted program (e.g., Zeidner and Shani-Zinovich, 2015). However, this relationship clearly requires more research.

#### Between-Group Differences

High self-esteem depends on high SC; yet the extent to which each facet contributes to self-esteem may vary. We have sketched the theoretical mechanisms in the previous paragraph; in the following, we will briefly outline empirical findings on differences in SCs and self-esteem, differentiated by gender and achievement tracks, as this will be the focus of our analyses to come.

Considering the developmental tasks of adolescence, all three facets—academic, physical, and social SC—can be expected to make important contributions to self-esteem. Earlier findings suggested a strong influence of appearance SC, which is understandable given the salience of bodily changes in the context of developmental tasks (e.g., Marsh, 1986; Harter, 1999). Adolescents’ increasing autonomy affects both their social SCs (i.e., peer relations become more important than parent relationships) and academic SC (as achievement is the social norm of the “adult” world, it is conceivable that its importance decreases especially when adolescents want to distinguish themselves from the adult world).

##### Gender

With gender roles becoming more salient in adolescence, it can be assumed that though appearance SC is important to all adolescents, girls’ self-esteem is likely more affected by low appearance SC. Meta-analytic results reveal absolute gender differences in all self-related constructs. Boys score higher than girls in self-esteem (reviews by Robins et al., 2002; Hyde, 2005), and while boys’ self-esteem increases, girls’ self-esteem decreases over time (Block and Robins, 1993). For body esteem, medium effects were observed, with a trend toward increasing differences (Feingold and Mazzella, 1998). Furthermore, high-achieving girls still struggle to integrate success into their feminine self-image (Skelton et al., 2010). Interestingly, meta-analytic findings including year of publication reveal that boys’ appearance SC became significantly higher than girls’ after 1980 only (Gentile et al., 2009).

##### Achievement

As outlined above, self-related constructs hinge on one’s frame of reference. While academic SC depends on individual achievement and vice versa, as described in the reciprocal effects model (e.g., Marsh and Martin, 2011), the frame of reference may increase (“basking in reflected glory” effect; Cialdini et al., 1976) or decrease academic SC (“big-fish-little-pond” effect; e.g., Marsh et al., 2008). Usually, frame-of-reference effects are observed at the classroom level. However, they may also extend to the overall track (especially considering the stigma attached to attending the lowest secondary or even the special education tracks; Whitley, 2008; Knigge, 2009). Though solid evidence on the relative contributions of individual ability, classroom, and track to SC is yet lacking, some evidence comes from giftedness research, where ability grouping is used to foster individual development. With the exception of gifted underachievers, gifted students’ self-esteem is somewhat higher than that of their average-ability peers. Higher intelligence increases the likelihood for high achievement, which influences academic SC in turn. Meta-analytic findings show that the overall higher self-esteem of gifted achievers is largely due to their higher academic SC (Hoge and Renzulli, 1993). As indicated above, ability grouping of gifted students affects their academic SC negatively when groups have been newly formed. Findings on social SC are mixed: Some studies found no effects on parent or peer SC (Marsh et al., 1995), while others identified small differences favoring students attending gifted programs (Marsh et al., 2001) or, longitudinally, positive (but not sustainable) effects of ability grouping on students’ social SC of acceptance (Vogl and Preckel, 2014), and still others identified lower social SC in accelerated gifted students (with effects dissipating for girls, but not for boys, after 2 years; Hoogeveen et al., 2009). There is even less evidence for differences in physical or appearance SC: Marsh et al. (1995) found no differences in physical or appearance SC in students attending a gifted program. In contrast, Brounstein et al. (1991) found lower appearance SC scores in gifted students; similar findings were reported by Veiga (2009), who identified higher appearance SC in low compared to high achievers. In sum, the questions how the different SCs are interrelated, what role achievement level plays in the process, and whether compensatory mechanisms between the different SCs are at work are still far from explored.

### Developmental Tasks of Adolescence

The ideal norms of success, beauty, and popularity are not only reflected in people’s SCs but translate into expectations toward individuals who are growing into adulthood. These developmental tasks can be considered “jobs” one has to accomplish to proceed successfully to the next developmental stage (Havighurst, 1948). Developmental tasks are driven by biological changes and changing expectations of society resulting from them. Adolescents, who find themselves at the transition between childhood and adulthood, need to accomplish several developmental tasks that relate to the three domains outlined above. According to Fend (2003), developmental tasks of adolescence can be divided into three domains, which parallel the three dimensions of the “ideal person” and the three main facets of SC: (i) the cultural/criterial domain (graduating from high school, choosing a career path that suits and eventually feeds them), which relates to success and academic SC; (ii) the intrapersonal domain (including coming to terms with bodily changes), which relates to appearance and physical SC; and (iii) the interpersonal domain (making close friends, finding a mate, and also renegotiate relationships with adults), which relates to popularity and social SC.

School is an important developmental context where adolescents are confronted with these developmental tasks, yet to different extents. Cognitive learning goals are still considered the most important outcomes of school, and although social-emotional learning outcomes have gained some importance (see Shavelson et al., 1976, for a brief outline of the historical trajectory), the development seems to have come to a standstill (or is even moving into the opposite direction by now). In the following, we will outline in how far SC and self-esteem are important in the school curriculum and then extend our perspective toward those societal changes that we consider most important with respect to the Triad of Unhappiness, with a focus on Germany, where the present study was conducted.

### The Importance of SC and Self-esteem in the School Curriculum

Schools as places of learning have focused on cognitive learning goals and achievement for the most of history, which is one likely reason why SC research (which is mostly conducted in schools) has focused so strongly on academic SC. Academic SC and achievement mutually influence each other (e.g., Marsh and Martin, 2011), which means that fostering either may lead to positive reciprocal effects and is, therefore, considered worthwhile. This is especially important in primary school, when children encounter systematic achievement-based comparisons for the first time and where teachers grade leniently (if at all) in order not to discourage children too early.

This cannot be stated for appearance SC, which plays hardly any role in the German school system. Physical education (PE) is about the only subject taught at all school types that might bear a relation to appearance SC. This is obvious for its components endurance, balance, flexibility, static strength, and explosive strength, but indirectly also for appearance (see Marsh and Redmayne, 1994, for an examination of the multidimensional physical SC), as physical activity supports the athletic, low body fat appearance representing the current beauty ideal. However, PE is usually one of the first subjects that is dropped when resources are scarce, underlining its low status as a non-academic subject (Marshal and Hardman, 2000). If body-related issues are addressed at all, this rather happens in terms of healthy nutrition or exercise. It is surprising that even the curricula of highly specialized subjects like Sports, Health, and Social Policy (a subject taught at intermediate secondary schools in all federal states examined in the present study; Ministry of Education, Research, and Cultural Affairs of Schleswig-Holstein, 1997; Ministry of Education and Cultural Affairs of Lower Saxony, 2007, 2011; Free and Hanseatic City of Hamburg, School and Vocation Office, 2011a,b) do not even mention appearance or appearance SC. We therefore conclude that appearance SC plays no role whatsoever in the German school curriculum, and that, consequently, systematic school-based interventions to increase appearance SC are lacking.

In contrast, social–emotional development is gaining importance. Social abilities are considered one important pillar of Weinert’s (2001) definition of competence, as they support students’ problem solving in variable situations. Team-working abilities play a particular role here. However, as described above for appearance SC, enhancing social SCs is not a curricular goal in itself. Lack of social competence is usually remedied by skill development (training social skills) rather than self-enhancement (increasing one’s social SC).

In the context of socio-emotional development as well, self-esteem as an emotional learning goal is nowadays considered an important outcome, too: first as an end in itself, but also as a means to high achievement. High self-esteem is related to positive beliefs students hold about themselves and thus to their wellbeing (see Baumeister et al., 2003, for an overview). In the school context, self-esteem is particularly important when it comes to failure (a necessary side effect of learning). McFarlin and Blascovich (1981) found that emerging adults form their expectations about future performance based on their chronic self-esteem rather than based on current performance feedback, which underlines the importance of high self-esteem.

In conclusion, school curricula target academic SC and self-esteem, the latter partly as a means in itself, partly in support of achievement development, whereas physical/appearance and social SCs play a minor role—if any at all.

### The Times, They Are A-Changing

#### Indicators for a Changing Focus on Achievement

That academic achievement is one ultimate goal of school does not come as a surprise, as education is an important resource in times of economic uncertainty. This uncertainty results in competition for the highest possible qualifications and fear of being left behind. Consequently, more students than ever attend the highest achievement tracks and enter tertiary education. According to the German Federal Bureau of Statistics (2016a), 41% of the German primary school students transitioned to the highest, 15% to comprehensive, and a mere 8% to the lowest secondary track in the academic year 2014/2015. The percentage of students transitioning to the highest track has increased by 5% since 2004/2005. In 2014, 53% of the 18- to 20-year-old age cohort acquired a college or university entrance qualification, compared to 49% in 2010 (Federal Bureau of Statistics, 2015a). Between 2000 and 2015, the number of people entering tertiary education has increased from 314,956 to 503,630 (Federal Bureau of Statistics, 2016b). In contrast, the number of students on the lowest secondary track had decreased from 21 to 12% between the academic years 2004/05 and 2014/15 (Federal Bureau of Statistics, 2016a). In some German federal states, this has resulted in “lower-track comprehensive schools” that integrate low and intermediate secondary tracks (e.g., “regional schools” in Rhineland-Palatinate).

Depending on the German federal state one lives in, students take 8 or 9 years to complete the highest secondary school degree. After the introduction of the 8-year highest track (“G8”), which had led to great dissatisfaction in students and parents, several federal states now offer students the choice between G8 and G9 (9-year highest track). Earlier findings indicated that shortening secondary school by one year would benefit the top 25% only, yet the remaining 75% would fare better with nine years of secondary schooling (Heller, 2002). In states where the G8 has remained, the majority of students are thus facing extreme pressure to achieve. Those struggling with demands exceeding their ability level often benefit from private tutoring, which has become a large economic factor. Estimates of the average sum spent per student and year in Germany range from 108 to 168€ (Statista, 2016a). A study by the Bertelsmann foundation, based on data from the 2003 Program for International Student Assessment (PISA) and the 2006 Progress in International Reading Literacy Study (PIRLS) data, showed that 15% of the PISA participants made use of commercial lessons, with those attending the highest track secondary school representing the largest group (Klemm and Klemm, 2010). Schneider (2004) points out that, interestingly, the target group for private tutoring is by far not limited to weak students, but also includes strong students who want to become even better, corroborating the assumption that competition is getting tougher. Though the 15% of the 15-year-old German PISA participants who received tutoring was still substantially below the average of 26.3% of the Organization for Economic Cooperation and Development (OECD), some countries have ratios as low as 2.4% (Finland; Prenzel et al., 2004), indicating that the German educational system cannot fully absorb the learning needs of its increasingly heterogeneous student population. Eventually, this may exacerbate educational disparities for families who cannot afford private lessons. As actual figures depend on the operationalization of tutoring, it is difficult to compare studies over time and thus to pinpoint trends. Yet with the importance of higher degrees and the increasing percentage of students attending higher tracks, it is likely that those parents who can afford it will try to help their offspring achieve at the highest possible level.

One further indicator for an increasing focus on professional appearance and behavior is adolescents’ use of professional online communities. Five percent of German teenagers age 14 and above use platforms like LinkedIn or XING (Busemann, 2013; private online communities are discussed below). In sum, we are observing a trend toward higher formal qualifications and an early career focus, along with an increasing pressure to achieve.

#### Indicators for a Changing Focus on Appearance

We are surrounded by beautiful people—at least if we believe the media, which portray good-looking (and usually heavily photoshopped) individuals regardless of the product being advertised. The number of opportunities to feel dissatisfied with one’s appearance is growing. Between 1985 and 2014, US-Americans’ daily per capita exposure to media increased from 433 to 590 minutes. The number of advertisements US-Americans have the chance to see or hear during that time has grown from 296 to 362, and the number of advertisements that actually grasped their attention for at least a few seconds has increased from 121 to 153 during the same time span (Media Dynamics Inc, 2014). This amounts to about ten consciously perceived ads per waking hour on average.

Appearance and products and services related to it are an important economic factor. In Germany alone, the volume of sales for decorative cosmetics has risen from 1.15 to 1.56 billion Euro between 2004 and 2015 (Statista, 2016b). According to the ISAPS International Survey on Aesthetic/Cosmetic Procedures, the number of cosmetic surgeries has increased by 43% between 2010 and 2014, totalling 9,645,395 surgical interventions worldwide (ISAPS, 2013, 2015). This trend is evident in men, too. For example, the percentage of men under 30 who place a high importance on their looks has increased from 43 to 56% between 1990 and 2011 (Schneller, 2011). The number of male breast reduction surgeries in the USA has increased by 173% between 1997 and 2015 (ASAPS, 2015). These realities likely influence people’s awareness of themselves and how they feel about their looks, i.e., their appearance SCs. For instance, in a study using comparable samples across an interval of 30 years, women reported more negative attitudes toward their bodies in 1996, compared to 1966 (no similar differences were found for men; Sondhaus et al., 2001).

The increasing focus on looks affects adolescents, too: 24% of German students age 10–17 report spending money on body care (42% of the girls, 8% of the boys, percentages increasing with age; Lange and Fries, 2006). The 2010 “Trend Tracking Kids” study estimated that German 6-to 19-year olds spent 947 million Euro on body and hair care, cosmetics, hairdressers, and solarium visits (Iconkids and youth, 2010). A recent representative online study by the industrial association for body care and detergents (Industrieverband Körperpflege und Waschmittel [IKW], 2016), conducted by an independent research institute, found that body and beauty care are important to 73% of over 1000 adolescents and young adults between 14 and 21 years. 85% use cosmetics to feel more secure. 62% of the girls use mascara daily (Industrieverband Körperpflege und Waschmittel [IKW], 2016). A study from the late 1990s showed that those adolescents spending most on their looks are more likely from lower and lower-middle class backgrounds and place much importance on brands, indicating compensatory consumption behaviors; this is especially true for girls (Lange, 1997). We will get back to this point later.

In sum, we are observing an increasing use of appearance-related products, procedures, and services, which represent an important market segment. The growing prevalence of advertisements (and, thus, idealized people in them) likely contributes its share to this trend.

#### Indicators for a Changing Focus on Social Relationships

The above-described tendencies to shorten secondary schooling time at the highest track and the increasing pressure associated with it decrease teenagers’ time to simply “chill out” with friends. On average, German children and adolescents work more than 38.5 hours per week in or for school (UNICEF, 2012), which is comparable to an adult’s full-time job. With increasing age, academic workload rises to up to 45 hours per week in Grades 9–13, resulting in less time for other activities (e.g., family time, playing with friends, “chilling out”, or other hobbies; self-reported data, UNICEF, 2012). On average, children and adolescents spend 18 h per week with their families, thus more than with friends (up to 12 h per week). Though meeting peers in person is still the most important real-world activity of the German 12- to 19-year olds (78% meet friends daily or several times a week; Medienpädagogischer Forschungsverbund Südwest, 2015), the percentage has substantially decreased, compared to 88% a decade earlier (Medienpädagogischer Forschungsverbund Südwest, 2005). This is in line with meta-analytic findings from developmental psychology, which reveal an extension of friendship networks during adolescence and young adulthood (Wrzus et al., 2013), whereas the size of family networks remains quite stable over the lifespan.

Besides school-related changes, we observe a tendency toward education-related leisure activities like music lessons or sports. A representative study based on the SOEP panel indicates that these activities play a role for 60% of the 16-year olds, compared to 48% in 2003 (Hille et al., 2013), which decreases time for friends and family. These findings can be interpreted in line with the increasing focus on an early professional appearance mentioned above.

One important change during the last decades is that more and more activities, including social interactions, occur online. Eighty-seven percent of German adolescents from 14 to 19 years used private online communities in 2013, compared to 40% in 2007; they represent the strongest age group (overall usage in 2013: 46%; Busemann, 2013). Social interactions are the most important activity in online communities. 85% of the community users send messages, 65% chat, and 64% like others’ content at least several times a week. 55% of US teenagers under 18 consider social networking sites like Facebook, Twitter, and Google+ (very) important for maintaining social relationships (Center for the Digital Future, 2015). However, social media may also be used for antisocial purposes. Like traditional bullying, cyberbullying (harming or harassing a person through electronic media) affects students’ self-esteem and psychosocial adjustment negatively (e.g., Katzer et al., 2009; Kowalski and Limber, 2013). It may even cause greater damage, as it happens (i) anonymously, (ii) publicly, i.e., with a larger potential audience, (iii) with fewer time or space constraints, (iv) with less direct feedback between bully and victim (Slonje and Smith, 2008), with less supervision (Patchin and Hinduja, 2006), and also with lasting effects, as the Internet does not forget (which becomes especially problematic in the case of sexting or happy slapping). More than half (52%) of the under 18-year-olds in the US know someone who has been bullied or harassed online; 19% of the Internet users in this group had been cyberbullied online in 2014 (Center for the Digital Future, 2015). Indirectly, frequent online social media use may thus be related to lower self-esteem in the long run.

Across age groups, respondents surveyed for the 6th Digital Future Report (Center for the Digital Future, 2015) indicated that the Internet has (greatly) increased their contact with others, the trend being even more pronounced in 2014 than in 2007. Overall, the Internet seems to broaden individuals’ social roles and extend their horizons regardless of sex (Colley and Maltby, 2008). There is no evidence that the Internet makes people lonely; instead, it may even affect their lives positively (Amichai-Hamburger and Hayat, 2011). In contrast, for real-life relationships, the representative US General Social Survey (GSS) reveals that the number of close confidants has decreased from 2.94 to 2.08 between 1985 and 2004, and that intimate ties are more strongly based on family than before (McPherson et al., 2006; see also Paik and Sanchagrin (2013) for a discussion of systematic biases caused by interviewer effects in the GSS data). The 2015 Shell study shows that family is a “safe harbor” for adolescents, too. The vast majority (92%) get along with their parents; the percentage of adolescents describing their relationship with their parents as very good has even increased from 35 to 40% between 2010 and 2015 (Albert et al., 2010, 2015).

According to McPherson et al. (2006), the decreasing number of confidants may also indicate that people become more selective about whom to trust with important questions; thus, they may not necessary become more isolated. The latter is confirmed for younger adults and adolescents. Loneliness in college students has decreased rather than increased between 1978 and 2009; similar trends can be observed for high-school students between 1991 and 2012 (meta-analysis by Clark et al., 2015).

In sum, we are observing that students spend most of their time in and for school and that “real” time with friends’ decreases, whereas the importance of online-based relationships increases. Family and parent relationships are still important for children and adolescents.

### Aims and Hypotheses of the Present Study

Our goals were (i) to provide an up-to-date account of the quality and extent to which different SC facets contribute to student self-esteem, thereby (ii) identifying possible differences between (iia) genders and (iib) types of secondary schools. Thus, our study links societal trends to individuals’ representations of themselves, in line with other studies examining SCs across cohorts (e.g., Yan and Haihui, 2005). These questions were examined based on a large and highly diverse sample comprising 2,950 students from 5 different secondary school types from the highest track to special education schools, which were assessed using a recently validated measure of SC (Weber and Freund, 2016). Our findings will thus provide insights into (i) whether previous findings still apply to today’s secondary school students, (ii) whether international findings apply to Germany, and (iii) whether these earlier findings can be replicated using a recently published validated SC measure.

As outlined above, academic SC is strongly based on both actual achievement and one’s relative achievement in comparison to classmates (Marsh et al., 2008). Academic achievement is particularly valued by parents (Harter, 1993), which might in part explain adolescents’ anti-achievement attitudes, as their developmental task is to become more independent from their parents and thus question their value system. In line with James (1890/1963), the importance of academic SC should grow with increasing track levels. However, girls, who obtain better grades than boys (Steinmayr and Spinath, 2008; see also Duckworth and Seligman, 2006), might be in a conflicting role, as high achievement is still ambivalent with regard to gender roles (Skelton et al., 2010). In sum, the influence of academic SC on self-esteem should be rather low overall, but slightly higher for the higher tracks. Gender differences will be examined exploratorily.

In line with theoretical conceptions from developmental psychology, current trends in society, and prior empirical findings, we expected appearance SC to affect self-esteem substantially across genders and school types, and more so than any other SC facet. No track-based differences are hypothesized. Yet regarding gender stereotypes, which are far from overcome, we expect stronger effects for girls than for boys.

Positive social relationships can be considered an indicator of social support. As described above, students spend most of their time in and for school. Therefore, we assume, that classmates and teachers are important reference persons for students. As outlined above, peer and parent support are related to different aspects of SC, but both affect self-esteem. Though Harter (1993) did not specify the impact of positive teacher relationships, similar effects as for parents are expected. It may be speculated that in an achievement-oriented society, both might compensate for lower-track students’ low academic SC and thus make a relatively greater contribution to their self-esteem. However, as it is not clear to what extent the effect of school type overrides the effect of the classroom, this question will be examined exploratorily. Conversely, the role of peers should be fairly consistent across school types. Gender effects will be examined exploratorily as well.

## Materials and Methods

### Sample

The sample included 2,950 students (47% girls; aged 10–19 years; *M*_age_ = 13.9 years, and *SD* = 1.90) from 36 German public secondary schools (Grades 5–11) located in Lower Saxony, Hamburg, and Schleswig-Holstein. In Germany, every child must enroll in school by the age of 6 and complete at least 9 years of schooling. Up to Grade 4, all children attend a comprehensive elementary school in the three states examined. At the secondary school level, students are then separated into different achievement-based school tracks. By the end of Grade 4, parents and teachers evaluate the academic achievement of the children and decide which secondary school track is best suited for the child. Most German federal states offer four types of secondary school: low-ability track (*Hauptschule*), intermediate-ability track (*Realschule*), high-ability track (*Gymnasium*; this is the most academically challenging secondary school), and a comprehensive mixed-ability track (*Gesamtschule*), which accepts students of all ability levels. Students with disabilities may attend regular schools (mostly the low- or mixed-ability tracks) or special educational schools (*Förderschulen*). Tracks differ mainly with regard to academic level and curriculum. Students from all ability tracks are included in the present study: high-ability track, *n* = 1 006 (53% girls; *M*_age_ = 13.4 years, and *SD* = 1.88); middle-ability track, *n* = 833 (46% girls; *M*_age_ = 13.7 years, and *SD* = 2.03); mixed-ability track, *n* = 469 (44 % girls; *M*_age_ = 14.2 years, and *SD* = 1.34); low-ability track, *n* = 298 (41 % girls; *M*_age_ = 13.9 years, and *SD* = 2.00); and special educational school (with an emphasis on learning), *n* = 344 (38 % girls; *M*_age_ = 15.3 years, and *SD* = 1.32). Hence, the sample was sufficiently heterogeneous and quite representative with regard to the different school tracks in the German educational system.

### Materials

The various SC variables were assessed using a German-language questionnaire specifically developed for both lower secondary students from special education schools (with an emphasis on learning) and from regular school types. The theoretical foundation for its construction and the empirical evidence for reliability and validity of the instrument are presented in Weber and Freund (2016). The questionnaire features eight subscales: SC in German (e.g., “I am good at German”) and mathematics (e.g., “I am good at Math”), general academic SC (e.g., “I am good at school”), parent relations (e.g., “I like my parents”), teacher relations (e.g., “I like most of my teachers”), classmate relations (e.g., “I like most of my classmates”), physical appearance (e.g., “I am good-looking”), and global self-esteem (e.g., “I like myself the way I am”). Appearance SC and self-esteem are represented by four items, whereas the other six subscales are represented by five items each. All items are positively worded. The 38 items are given as statements. Respondents indicate the degree of their agreement on a 4-point rating scale (1 = *no*, 2 = *rather no*, 3 = *rather yes*, and 4 = *yes*), with higher scores representing a higher SC. The original German version is available on request from the corresponding author. To minimize social desirability bias or any bias associated with self-reports, students had the additional option *do not know* (considered a missing value) if they did not want to answer the question or did not know what to choose.

### Procedure

The survey was conducted in class during school hours by the second author and trained test administrators in groups of 3 (special educational school) to 26 (high-ability track) students. Teachers were not present. As the university where the present study was carried out does not provide a standardized IRB approval procedure, we strictly adhered to the recommendations and guidelines of the local educational authorities, which are based on extensive experience with the population in question. As students under the age of 18 are only allowed to participate in surveys with their parents’ consent, parental consent was obtained prior to data collection, and only students with written approval by a parent or a guardian participated in our study. The response rate of the parental consent forms ranged from approximately 90% (high- and middle-ability tracks) to 50% (special educational schools). Students who had no signed parental consent or did not want to participate left the classroom during the survey. At the beginning of the survey, students were given an overview of the investigation and were guaranteed both anonymity of their responses and the freedom to drop out of the examination at any time for whatever reason. All students participated voluntarily. The standardized instructions were read aloud by the test administrators and students were asked to complete the questionnaire. Answering the questionnaire took between 5 and 25 min, with students at special educational schools taking the longest time. Students indicated their gender, date of birth, grade level, and school type.

## Results

### Descriptive Information and Correlations

**Table 1** reports correlations for the eight subscales as well as estimates of Cronbach’s α, means and standard deviations. Internal consistencies were generally high (α ≥ 0.82). Self-esteem was correlated with all seven SC facets, but especially with appearance SC (*r* = 0.74; *p* < 0.01). General academic SC was significantly associated with SCs in German (*r* = 0.52; *p* < 0.01) and mathematics (*r* = 0.57; *p* < 0.01). Boys showed higher self-esteem (*d* = -0.27) and appearance (*d* = -0.33) and mathematics SCs (*d* = -0.37), whereas girls had a higher SC in German (*d* = -0.24). Social SC facets and general academic SC showed none or only very small gender differences. Across the different ability tracks, students from the low-ability track and the special educational school had a higher self-esteem, a higher general academic SC, and a higher SC of teacher relations (**Table 1**).

**Table 1:** Intercorrelations (total sample), Cronbach’s alpha (α), mean (*M*), and standard deviations (*SD*) of the SC scales for the total sample (*N* = 2950), for girls (*n* = 1376) and boys (*n* = 1574) and for the different ability tracks.

Intercorrelations								Total			Girls			Boys		
			
	SE	AS	GA	GE	MA	PR	CR	α	*M*	*SD*	α	*M*	*SD*	α	*M*	*SD*
SE								0.86	3.24	0.69	0.89	3.09	0.74	0.82	3.36	0.62
AS	0.74							0.91	3.05	0.79	0.92	2.87	0.84	0.88	3.20	0.71
GA	0.40	0.26						0.87	2.89	0.62	0.88	2.86	0.62	0.85	2.92	0.61
GE	0.21	0.14	0.52					0.89	2.76	0.70	0.89	2.86	0.69	0.88	2.68	0.71
MA	0.30	0.19	0.57	0.07				0.94	2.81	0.89	0.94	2.62	0.91	0.93	2.98	0.85
PR	0.39	0.27	0.20	0.17	0.10			0.87	3.68	0.54	0.88	3.67	0.57	0.86	3.69	0.52
CR	0.37	0.33	0.19	0.15	0.12	0.23		0.86	3.37	0.60	0.87	3.36	0.62	0.85	3.38	0.59
TR	0.32	0.21	0.35	0.30	0.21	0.27	0.32	0.87	3.15	0.68	0.87	3.20	0.65	0.88	3.11	0.71

	**High**			**Intermediate**			**Comprehensive**			**Low**			**Special**		
					
	**α**	***M***	***SD***	**α**	***M***	***SD***	**α**	***M***	***SD***	**α**	***M***	***SD***	**α**	***M***	***SD***

SE	0.87	3.25	0.65	0.88	3.20	0.70	0.87	3.15	0.71	0.82	3.34	0.67	0.83	3.29	0.75
AS	0.90	3.05	0.71	0.92	3.04	0.81	0.91	2.98	0.80	0.86	3.17	0.80	0.91	3.03	0.90
GA	0.88	2.90	0.58	0.86	2.82	0.60	0.89	2.86	0.65	0.83	3.02	0.62	0.84	3.00	0.69
GE	0.89	2.79	0.67	0.88	2.61	0.68	0.89	2.78	0.70	0.85	2.91	0.70	0.91	2.88	0.81
MA	0.94	2.80	0.86	0.95	2.85	0.91	0.94	2.69	0.91	0.92	2.89	0.88	0.93	2.83	0.94
PR	0.84	3.72	0.49	0.85	3.69	0.51	0.89	3.55	0.63	0.83	3.77	0.45	0.93	3.65	0.66
CR	0.84	3.42	0.53	0.90	3.34	0.64	0.85	3.29	0.62	0.87	3.30	0.68	0.83	3.44	0.61
TR	0.87	3.13	0.65	0.87	3.10	0.68	0.89	3.06	0.71	0.84	3.28	0.67	0.87	3.38	0.69


*All correlation coefficients were significant at *p* < 0.01. High, high-ability track (*n* = 1006); intermediate, intermediate-ability track (*n* = 833); Comprehensive, mixed-ability track (*n* = 469); Low, low-ability track (*n* = 298); Special, special educational school (*n* = 344). SE, self-esteem; AS, appearance SC; GA, general academic SC; GE, German; MA, mathematics; PR, parent relations; CR, classmate relations; TR, teacher relations.*

### Measurement Invariance

To ensure that results are comparable across groups, measurement invariance needs to be ascertained (cf. Baudson et al., 2015). In a first step, we specified an 8-factor model (with each subscale of the questionnaire as a separate factor) for the total sample. Next, we examined measurement invariance across genders (Models 2–5) and across different ability tracks (Models 6–9). To evaluate the fit of the competing models, we used the Comparative Fit Index (CFI), the Tucker-Lewis Index (TLI), and the Root Mean Square Error of Approximation (RMSEA). For categorical data, CFI and TLI values above 0.960 and RMSEA values below 0.050 are considered good fit (Yu, 2002). In addition, we report χ^2^ statistics and degrees of freedom (*df*). **Table 2** shows how well the models fit the data and how they compare to the baseline (i.e., configural) model. We used the CFI and RMSEA statistics for the invariance tests because the χ^2^ difference statistic is highly sensitive to large sample sizes (Cheung and Rensvold, 2002). Lack of invariance is represented by a decrease of 0.005 or more for the CFI and by an increase in RMSEA by 0.010 or more (for more information about the cut-off criteria used in this study see Chen, 2007). Practical fit indices like CFI, TLI, and RMSEA remained similar or even improved slightly (**Table 2**). Therefore, measurement invariance across genders and ability tracks can be assured, such that meaningful comparisons among these groups are possible.

**Table 2:** Goodness-of-fit indices of the models.

Model	χ^2^	*df*	CFI	TLI	RMSEA	Model description
1	5861.118	637	0.970	0.967	0.053	8-factor model (total sample)
**Gender invariance**						
2	6071.528	1350	0.972	0.969	0.049	Configural
3	6135.698	1380	0.972	0.970	0.048	Weak
4	6349.468	1448	0.971	0.970	0.048	Strong
5	6315.692	1486	0.971	0.972	0.047	Strict
**Ability track invariance**						
6	7663.201	3185	0.973	0.971	0.049	Configural
7	7691.005	3305	0.974	0.972	0.047	Weak
8	8037.831	3577	0.973	0.974	0.046	Strong
9	8615.494	3729	0.971	0.973	0.047	Strict


*χ^*2*^, chi-square; *df*, degrees of freedom; CFI, Comparative Fit Index; TLI, Tucker-Lewis Index; RMSEA, root mean square error of approximation. All models are estimated by the weighted-least-square-mean-variance estimator (WLSMV) with theta-parameterization. All analyses conducted with M*plus* 7.1 (Muthén and Muthén, 1998–2013).*

### Latent Regression Analyses Predicting Self-esteem

After measurement invariance was ascertained, the next step was to examine the predictors of self-esteem separately for boys and girls and within each ability track. **Tables 3** and **4** show the unstandardized regression coefficients, standard errors, and the explained variance (*R*^2^) in self-esteem. All analyses were conducted with M*plus* 7.1 (Muthén and Muthén, 1998–2013). For both girls and boys, appearance SC was by far the most important predictor of self-esteem (girls: *B* = 0.82, *SE* = 0.02, and *p* < 0.01; boys: *B* = 0.70, *SE* = 0.07, and *p* < 0.01). For girls, appearance SC was slightly more important. Boys’ self-esteem was more strongly related to general academic and parent relations SCs than girls’ self-esteem (**Table 3**). Results furthermore demonstrate that self-esteem decreases with age in both girls and boys (girls: *B* = -0.11, *SE* = 0.01, and *p* < 0.01; boys: *B* = -0.05, *SE* = 0.01, and *p* < 0.01).

**Table 3:** Results of regression analyses predicting self-esteem for the total sample and for girls (*n* = 1323) and boys (*n* = 1495).

	Total		Girls		Boys	
			
	*B*	*SE*	*B*	*SE*	*B*	*SE*
AS	0.77^∗∗^	0.02	0.82^∗∗^	0.02	0.70^∗∗^	0.07
GA	0.21^∗∗^	0.05	0.12^∗^	0.05	0.33^∗∗^	0.07
GE	-0.08^∗∗^	0.03	-0.05	0.03	-0.14^∗∗^	0.04
MA	-0.01	0.03	0.02	0.03	-0.07	0.04
PR	0.14^∗∗^	0.02	0.10^∗∗^	0.02	0.20^∗∗^	0.04
TR	0.08^∗∗^	0.02	0.08^∗∗^	0.02	0.05^∗^	0.02
CR	0.03	0.02	0.03	0.03	0.03	0.03
Age	-0.08^∗∗^	0.01	-0.11^∗∗^	0.01	-0.05^∗∗^	0.01
Sex	0.41^∗∗^	0.04				
Intercepts^a^			0.00	0.00	0.07	0.27
*R*^2^	0.92		0.93		0.91	


*^a^In the female group, latent means were fixed to zero for identification purposes (reference group). *B*, unstandardized regression coefficient; *SE*, standard error; *R*^2^, variance explained by the seven SC facets; Age: years and months; Sex: female = 0, male = 1; total, total sample (*N* = 2818); AS, appearance SC; GA, general academic SC; GE, German; MA, mathematics; PR, parent relations; CR, classmate relations; TR, teacher relations. ^∗^*p* < 0.05 and ^∗∗^*p* < 0.01.*

**Table 4:** Results of regression analyses predicting self-esteem for the different ability tracks.

	High		Intermediate		Comprehensive		Low		Special Ed.	
					
	*B*	*SE*	*B*	*SE*	*B*	*SE*	*B*	*SE*	*B*	*SE*
AS	0.88^∗∗^	0.10	0.78^∗∗^	0.03	0.61^∗∗^	0.15	0.97^∗∗^	0.20	1.27^∗∗^	0.39
GA	0.05	0.05	0.39^∗∗^	0.09	0.22^∗∗^	0.06	0.28	0.20	0.54	0.65
GE	0.03	0.03	-0.16^∗∗^	0.05	-0.07^∗^	0.04	-0.17	0.11	-0.23	0.27
MA	0.06	0.03	-0.10	0.06	-0.01	0.03	0.02	0.07	-0.19	0.32
PR	0.13^∗∗^	0.03	0.21^∗∗^	0.03	0.13^∗^	0.06	0.33^∗^	0.14	0.00	0.06
TR	0.04	0.03	0.03	0.03	0.07	0.04	0.16^∗^	0.08	0.08	0.10
CR	0.03	0.03	0.00	0.03	0.03	0.06	-0.15	0.10	0.09	0.09
Age	-0.09^∗∗^	0.02	-0.11^∗∗^	0.02	-0.07^∗^	0.04	-0.07^∗^	0.04	0.07	0.07
Sex	0.30^∗∗^	0.06	0.36^∗∗^	0.07	0.58^∗∗^	0.13	0.30	0.16	0.96^∗∗^	0.32
Intercepts^a^	0.19	0.27	0.00	0.00	-0.22	0.55	0.12	0.64	-0.97	1.54
*R*^2^	0.93		0.92		0.97		0.98		0.90	


*^a^In the intermediate-ability group, latent means were fixed to zero for identification purposes (reference group). *B*, unstandardized regression coefficient; *SE*, standard error; *R*^2^, variance explained by the seven SC facets; Age: years and months; Sex: female = 0, male = 1; an High = high-ability track (*n* = 987); Intermediate, intermediate-ability track (*n* = 809); Comprehensive, mixed-ability track (*n* = 429); Low , low-ability track (*n* = 275); Special, special educational school (*n* = 318). AS, appearance SC; GA, general academic SC; GE, German; MA, Mathematics; PR, parent relations; CR, classmate relations; TR, teacher relations. Note that standard errors are in part unequal; especially for students at special educational schools. ^∗^*p* < 0.05 and ^∗∗^*p* < 0.01.*

For students attending special educational schools (area of learning), appearance SC was by far the most important significant predictor of self-esteem (*B* = 1.27, *SE* = 0.39, and *p* < 0.01). But also across the other ability tracks, appearance SC showed its predictive power for students’ self-esteem. Except for the high-ability track (*B* = 0.05, *SE* = 0.05, and n.s.), general academic SC was likewise an important predictor of self-esteem. Parent relations SC significantly predicted self-esteem for students at high-, middle-, mixed- and low-ability tracks, but not for students at special educational schools. For students at low-ability schools, teacher relations SC was a significant predictor, too (*B* = 0.16, *SE* = 0.08, and *p* < 0.05). Classmate relations SC does not seem to play a role for students’ self-esteem (total: *B* = 0.03, *SE* = 0.02, and n.s.). Across all subgroups, the seven SC facets strongly predicted students’ self-esteem (*R*^2^ = 0.90–0.98).

## Discussion

### Summary of the Findings

Across all subgroups of a highly diverse secondary school sample, we found that students’ self-esteem consistently hinges on how attractive they perceive themselves. Compared to these large effects, the role of academic and social SCs is much smaller. With regard to the “Triad of Unhappiness,” a subjective lack of attractiveness therefore has a much greater potential to make students unhappy, and other aspects of SC likely cannot fully compensate for low appearance SC. Absolute SC levels were similar across subsamples, indicating frame-of-reference effects. Girls’ self-esteem was even more heavily influenced by how attractive they considered themselves than boys’, whereas academic SC was somewhat more important for boys, suggesting that gender stereotypes—beautiful women, successful men—may still be at work here.

The predictive power of different SC facets depended on the academic track students attended. Appearance SC was a strong predictor of self-esteem especially for special education students, whereas the self-esteem of students in the highest track did not hinge on their academic SC, suggesting that frame-of reference effects extend across school types, too. Teacher relations affected self-esteem of both genders. However, looking into the different tracks, only self-esteem of students in the low-ability track were influenced by how well they got along with their teachers. Parent relations contributed to self-esteem in all groups except for special education students. In line with previous findings, SE also decreased with age; we will discuss this finding below.

### Strengths and Limitations

Our study comprises a large and diverse sample from three German federal states, covering the most important secondary school types, which, across Germany, account for 67% of the student population (Federal Bureau of Statistics, 2015b). Despite the quality of the sample, data are not representative for all of Germany, much less worldwide; as the education sector is governed by each the federal state individually (resulting, strictly speaking, in 16 different German education systems), this may limit the generalizability of our findings.

The scales we used have been thoroughly validated (Weber and Freund, 2016) and comprise a large number of SC facets, thus allowing to assess the construct and its facets in a highly differentiated way. While there are empirically validated German versions of the Self Description Questionnaire (SDQ) I (Arens et al., 2011, 2013) and the SDQ III (Schwanzer et al., 2005), the SDQ II (which is designed for adolescents) has so far not been translated into German. However, the questionnaire we used is based on the scales of the Self Descriptions Questionnaires (see Marsh, 1990, for an overview) and the theoretical model of Shavelson et al. (1976). The advantage of our instrument consists in its economic usability (with 38 items, the questionnaire we used is much more economic than the 102-item SDQ II). Moreover, this new instrument allowed us to investigate whether the SCs of teacher and classmate relations affected students’ self esteem, as these scales were newly developed by Weber and Freund (2016) and do not have an equivalent in the SDQs). However, the PSC scale is limited to self-perceived attractiveness. Including other aspects, e.g., physical ability SC, might allow to expand on the findings presented here, and also to examine the interactive and discrepancy models (cf. Marsh, 1986) across school types.

### Tentative Explanations for the Findings

The finding that girls’ self-esteem hinges more strongly on appearance SC than boys’, whereas boys’ self-esteem is more influenced by general academic SC than girls’, suggests that gender stereotypes still prevail. Adolescence is a period of identity formation; this also includes sexual and gender identity. Stereotypes (e.g., that women should be attractive and men successful) may provide an orientation in the process of finding one’s individual answer to the question what it means to be a woman or a man. Still, in absolute terms, both sexes suffer from feeling unattractive.

The fact that appearance SC was a particularly strong predictor for special education students, and that general academic SC was not an important predictor for students in the highest track, suggests two different mechanisms, which may be related to students’ internalization of how others view them. Identity development is the crucial developmental task in adolescence (Harter, 1999) and results from the constant interaction and negotiation between the inside and the outside view of an individual (the “I” and the “Me”; James, 1890/1963); therefore, we may speculate that others’ perspective on the group one belongs to gains particular importance during adolescence.

In this context, the concept of the imaginary audience may also shed some light on the findings, which describes the phenomenon that “the adolescent … fails to differentiate between the objects toward which the thoughts of others are directed and those which are the focus of his own concern. … Accordingly, … he assumes that other people are as obsessed with his behavior and appearance as he is himself.” (Elkind, 1967, p. 344). As one’s appearance is the first thing others perceive, this may explain the importance of one’s self-perceived looks for overall self-esteem we identified here. Our finding that the influence is even stronger for girls is in line with most findings on gender differences in imaginary audience, though there are contradicting results as well (see Galanaki, 2012, for an overview). However, the imaginary audience does not explain the age-related decline in self-esteem. For instance, Ryan and Kuczkowski (1994) found that imaginary audience scores (especially the Abiding Self subscale, which describes aspects of one’s personality the adolescent perceives as stable, e.g., abilities or personality traits) decline somewhen between Grade 9 and 12. As they are negatively related to global self-esteem, an age-related increase in self-esteem should be expected. Thus, other factors are likely at work here, such as increasing outside (and subjective) pressure relating to achievement- and partnership-related developmental tasks. However, these ideas are rather speculative at the present stage and would therefore require further empirical examination.

The outside view also comprises stereotypes that are prevalent in society, which might help explain observed differences between school types. Hence, it is conceivable that special education students, who are ascribed low academic and social competencies (e.g., Whitley, 2008), emphasize the one aspect where they stand a chance to be superior (or at least equal) to others. In contrast, students attending the highest track have a rather positive image, possibly allowing them to neglect the one aspect where their superiority is undisputed.

Considering different aspects of social SC, the beneficial effects of a positive relationship with one’s teacher for low-track secondary school students may be due to the fact that these students are often considered “difficult,” thus requiring closer attention from their teachers than in other tracks. The teacher-student relationship may thereby become particularly salient. This interpretation is supported by the fact that the second-highest coefficient was observed for special education students, who also require much attention. In addition, teachers can be considered representatives of an achievement-oriented society. Unlike students at special education schools, those on the lowest academic track are still considered part of the regular school system. In an achievement-oriented society, attending the low track may represent an ego threat and thus a particular challenges for these students’ self-esteem. Being acknowledged by someone who represents society’s achievement-oriented values may therefore buffer some of the negative effects of attending the low-achieving track.

The finding that the parent relationship had no effect on special education students’ self-esteem is puzzling. It is possible that this finding might be due to the sample, as it became clear in discussions during data collection that many of the special education students were raised in children’s homes, foster care, or by single parents. It is conceivable that the “loss” of one or more parents may have affected the link between parent relationship and self-esteem. A systematic evaluation of this post-hoc explanation would require that future studies gather systematic data on students’ family background.

### Why Is Appearance SC So Neglected?

In part, these results confirm that physical SC, and especially appearance SC, is still the most important predictor of self-esteem (e.g., Harter, 1993). Compared to Marsh’s (1986) results, which revealed a strong impact on social SC on self-esteem, our findings indicate that the importance of peers has much decreased nowadays. With respect to the extant clinical literature on body image and its relation to self-esteem, it is surprising that, apparently, education has cared so little about what the substantial effects of appearance SC on self-esteem imply for practice. Considering the number of publications on the different SC facets, academic SC seems to have a much better lobby than other SC components. One tentative explanation lies in the ancient body/mind distinction (which, as we know today, is much less clear-cut than former generations used to think). Since Aristotle, the body has been considered inferior to the mind and had to be “civilized” through mental powers. All things physical are considered ephemeral, whereas the mind only is able to create something beyond an individual’s worldly existence.

The mind-body hierarchy is reflected in school, too. Although more recent curricula stress that school should consider the holistic development of all students, this is not yet fully reflected in practice. On the contrary, there are numerous examples for the neglect of the body in the schools. For instance, whenever resources are scarce, PE is one of the first subjects to suffer from budget cuts; generally, PE is anything but in good shape (Marshal and Hardman, 2000). Large-scale studies like PISA or PIRLS, which, by now, are quite established in the school system, have exclusively focused on students’ academic achievement up to now.

### Outlook: Consequences for Research, Teaching and Learning

As the limitations of one study always imply opportunities for future research, attempts to generalize our findings, e.g., to different countries and across cultures, may be undertaken. With increasing globalization, it is also of interest to examine SC development across cultures longitudinally to find out whether increasingly similar cultural influences (mainly Western-dominated) also lead to similar SCs in adolescents or whether effects may be different (e.g., a greater importance of the social SCs in more collectivist cultures). Our study combined observations on cultural and societal changes pertaining to achievement, popularity, and appearance with developmental tasks and SC in corresponding domains. This line of research might be meaningfully expanded by examining how students perceive their changing environments—e.g., in how far the frequent exposure to perfect looks through mass media directly affects their self-concepts and self-esteem over time, thus linking research on media influence and self-concept research. Furthermore, one particularly interesting extension of our study would be to relate students’ self-concepts to actual data on their achievement, popularity, and attractiveness, thus updating and expanding on Marsh’s (1986) findings.

Considering the positive impact of exercise on student health, body composition, appearance SC, and thus self-esteem, cutting down PE in schools is the wrong way. Instead, one might rethink the concept of PE overall. Instead of providing another subject where performance is graded, an alternative approach might be to counterbalance the cognitive focus of other subjects by providing an opportunity to enjoy exercise in a playful way. Children are intrinsically motivated to keep moving; conserving this activity level by making exercise fun and enjoyable might therefore have long-term outcomes on health even beyond school.

Furthermore, health in general should play a more important role in schools, e.g., by offering subjects concerned with healthy eating, but also wellbeing and stress reduction in general. Currently, socio-emotional learning goals such as self-esteem are empty shells rather than practical reality. A stronger focus on self-acceptance and individual development in line with learning rather than performance goals may also have the positive side effect of reducing competition and increasing cooperation between students. The “Triad of Unhappiness” may offer a framework for critical discussions of which domains are considered compatible and which are not.

A critical reflection of appearance, beauty, and attractiveness is clearly missing in the academic curriculum considering the importance of appearance SC for students’ wellbeing. Though it may be tempting, the response should not be to devalue students’ attempts to look good as shallow. We think that rather than ignoring the importance of appearance, curricula should take this strong concern of students seriously and foster critical reflection. In doing so, the curriculum should stress that beauty is a social construct and is therefore inseparable of its historical, social, medial, and economical context. This might help decrease the pressure students are facing, and which all too often impedes their positive development and individual growth.

## Author Contributions

TB came up with the story and wrote the introduction and discussion sections. KW developed the instrument, collected and analyzed the data, and wrote the methods and results sections. PF contributed to data analysis and interpretation of the findings.

## Conflict of Interest Statement

The authors declare that the research was conducted in the absence of any commercial or financial relationships that could be construed as a potential conflict of interest.

## Footnotes

1 For reasons of conclusiveness, our focus is on the Marsh/Shavelson approach here, as the questionnaire we used is based on this model. Readers interested in alternative approaches to the integration of cognitive and affective aspects of self-esteem are referred to Mruk (2013), who provides an overview of the debate as well as a theoretical integration into a two-factor model. We thank Reviewer 1 for pointing this out.
